# Supplementary material for: Mutations in ELAC2 associated with hypertrophic cardiomyopathy impair mitochondrial tRNA 3′‐end processing
Source: Hum Mutat. 2019 Jun 18;40(10):1731–48. doi: 10.1002/humu.23777 (PMC6764886; doi:10.1002/humu.23777)
Supplement: Supplementary file 2 — Supporting information [file HUMU-40-1731-s002.pdf]

SUPPLEMENTARY INFORMATION

Mutations in *ELAC2* associated with hypertrophic cardiomyopathy impair mitochondrial tRNA 3'-end processing

Saoura M, Powell CA *et al*.

CONTENT:

1. **SUPPLEMENTARY PATIENT INFORMATION – page 2-15**
2. **SUPPLEMENTARY FIGURES S1-S9 – page 16-25**
3. **SUPPLEMENTARY REFERENCES – page 26**

Patient 1
c.202C>T; c.1478C>T (p.Arg68Trp; p.Pro493Leu)

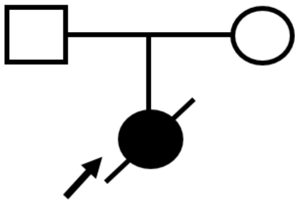

Patient 1 was a female, the first child of unrelated, non-consanguineous German parents. She developed severe muscle weakness, respiratory insufficiency and cardiomyopathy with lactic acidosis in the newborn period, with rapid progression leading to death at 3 weeks of age. Histolopathological examination of a skeletal muscle biopsy showed COX-deficient/SDH-hyper-reactive fibres, whilst biochemical analysis of respiratory chain enzyme activities revealed a severe combined respiratory chain defect involving complexes I and IV.

This patient has been previously published [1] where a p.Ala541Thr heterozygous variant was reported to present together with a second heterozygous c.1478C>T, p.Pro493Leu variant, suggesting a possible compound heterozygosity. Subsequent cDNA analysis of patient cells revealed the two variants being in cis and identified on the other allele a further heterozygous change (c.202C>T, p.Arg68Trp) which was confirmed in genomic DNA. The p.Arg68Trp variant is predicted to be damaging and absent from the gnomAD database and, as such, is a likely pathogenic variant; this has led to the functional consequence of this variant being tested in *vitro* (current manuscript).

Patient 2
c.2009del; c.1423+1G>A (p.Cys670Serfs*14; consensus splice mutation)

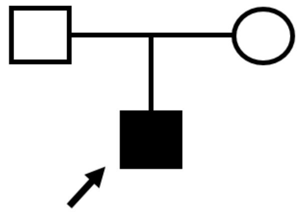

Patient 2 is an 8-year-old male, the second child of non-consanguineous Caucasian Australian parents. He was born at 39 weeks gestation after a pregnancy complicated by poor fetal growth. At birth he weighed 2.75 kg (3rd centile), but his length (47cm) and head circumference (33cm) were on the 10th centile. He fed poorly from the outset, requiring supplemental nasogastric tube feeding and was noted to be constipated. Concerns regarding his neurodevelopment were expressed from 9 months of age. Clinical examination at 8 years revealed short stature (height 112.5 cm; <1st centile) and microcephaly (head circumference 46.0 cm; <2nd centile) with dysmorphic facial characteristics including a high and broad forehead, prominent metopic suture, cup shaped ears with upturned lobules, epicanthic folds, down slanting palpebral fissures and a bulbous nasal tip. Tone was increased in his legs and he had truncal and limb ataxia. He had previous bilateral orchidopexies and surgical correction of bilateral strabismus.

The following investigations were performed and unless indicated were normal: brain MRI, nerve conduction studies, skeletal survey, urine amino and organic acids, transferrin isoforms, 7-dehydrocholesterol, leukocyte lysosomal enzymes, very long chain fatty acids, biotinidase, plasma lactate, CSF amino acids, CSF lactate, CSF glucose, CSF pterins and thyroid function tests. CSF lactate was 2.7 mmol/L (NR 1.2-2.1 mmol/L) and simultaneous paired plasma lactate was 2.8 mmol/L (NR 0.7-2.1 mmol/L). Creatinine kinase was mildly elevated (283 U/L; NR 25-200 U/L). Array CGH identified a 252 kb duplication of chromosome Xq22.1-22.2, approximately 100 kb upstream from the proteolipid protein 1 (*PLP1*) gene. This was also present in his asymptomatic grandfather, suggesting that the duplication is unlikely to be related to the patient's clinical features. A clinical exome NGS panel involving the sequencing and deletion/duplication analysis of 4,637 genes known to be associated with medically-significant conditions identified compound heterozygous *ELAC2* variants; a paternally-inherited c.2009del (p.Cys670Serfs*14) variant and a maternally-inherited c.1423+1G>A consensus splice site mutation.

Patient 3
c.297-2_297delinsTG; c.2342G>A (consensus splice mutation; p.Arg781His)

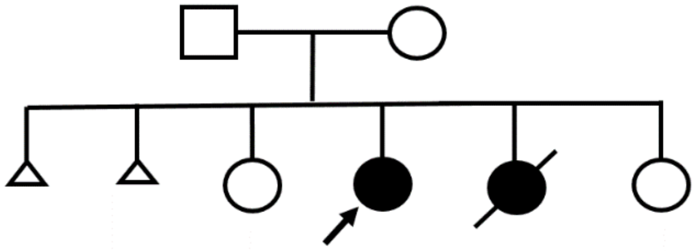

Patient 3 is the second daughter born to non-consanguineous Irish parents. She was born at term by normal delivery with a birth weight of 2.89 kg. There were parental concerns about developmental delay, decreased energy and irritability when she was 18 months old. Subsequent investigation at 2 years of age revealed mild global developmental delay, weight and length between 9th and 25th centiles and head circumference between 2nd and 9th centiles. Echocardiogram revealed a mild left ventricular (LV) hypertrophic cardiomyopathy (HCM). She had one elevated blood lactate level of 3 mmol/l (NR <2.2). Plasma amino acids showed intermittent elevation of alanine and proline. Urine organic acids were normal. Skeletal muscle biopsy showed type two fibre atrophy with occasional COX-deficient fibres, although mitochondrial respiratory chain studies of a skeletal muscle homogenate were reported to be normal. A mitochondrial disease gene panel revealed segregating *ELAC2* variants. She continues to show faltering growth with head circumference and length on the 2nd centile and weight on the 25th centile. She has persistent mild developmental delay; she walked at 26 months and had two-word phrases at age 2 years and at the age of 5 years continues to make progress. The family history is notable for two previous first-trimester miscarriages and a younger sibling death. Her younger sister died suddenly at 4 months of age and was noted to have HCM at autopsy, with histological studies suggestive of a mitochondrial cytopathy. An older sister and younger sister are both healthy.

Patient 4
c.2186A>G; c.2342G>A (p.Tyr729Cys; p.Arg781His)

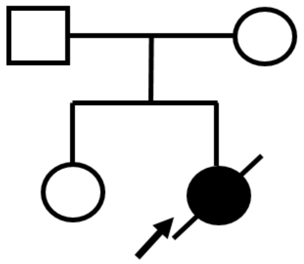

Patient 4 was the second daughter born to non-consanguineous Caucasian parents, who have an older, healthy 6 year old daughter. She was born at 34 weeks gestation and required supplemental oxygen until postnatal day 10. Following discharge she had apparently normal early development until presenting in acute cardiogenic shock at the age of 11 weeks. She had marked lactic acidosis, left ventricular hypertrophy (LVH), a pericardial effusion and poor systolic and diastolic function. Whilst initially supported with a pericardial drain and inotropes with a presumed diagnosis of myocarditis, all microbiological investigations for an infective cause proved negative and the child's cardiac function continued to deteriorate. She was successfully resuscitated following a cardiac arrest, but it became clear that her cardiomyopathy was irreversible and the family agreed to the withdrawal of intensive support following a diagnostic muscle biopsy at the age of 12 weeks. This biopsy demonstrated a severe and isolated loss of mitochondrial complex I activity. Post-mortem histology of cardiac muscle demonstrated focal myofibre disruption and deposition of lipid and membrane bound glycogen vacuoles.

Patient 5
c.460T>C; c.460T>C (p.Phe154Leu; p.Phe154Leu)

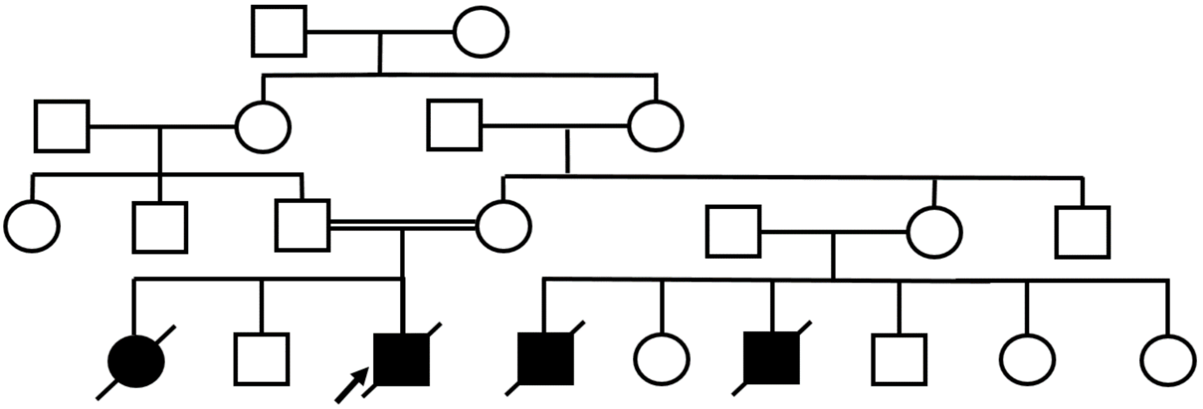

Patient 5, the index case in this family, is a male infant, the third child born to consanguineous, first cousin, Kuwaiti-Arab parents. He was born preterm at 36 weeks via caesarean section with a birth weight of 2.65 kg. He developed symptoms of tachypnoea, tachycardia and feeding difficulties within days of birth. On assessment, he was noted to have a grossly elevated blood lactate (12.3 mmol/L; NR 0.7-2.1 mmol/L) that responded to supportive fluid management. An echocardiogram confirmed HCM, progressing rapidly over subsequent months leading to complete heart failure and death at age 1 year. A homozygous p.Phe154Leu *ELAC2* variant was identified following whole exome sequencing (WES); parents were confirmed to be heterozygous carriers

The older sister of the index case was noted to have a Tetralogy of Fallot on antenatal scans. Following birth, this congenital cardiac defect was surgically ameliorated with a modified Blalock Taussig shunt and definitively corrected at 2.5 months old. An unexplained concentric biventricular cardiac hypertrophy had been noted on echocardiogram and did not resolve following surgery. Metabolic investigations revealed elevated urinary 2-oxyglutarate, succinate, fumarate and maleate with dicarboxylic and ethylmalonic acids also present in urine. Raised urinary 4-hydroxyphenyl lactate and 4-hydroxy pyruvate indicated some degree of liver dysfunction. Plasma lactate was elevated (4.4 mmol/L; NR 0.7-2.1 mmol/L) early in the course of her disease, though progressive heart failure may have been a contributory factor. Electron microscopy of myocardial biopsy revealed giant mitochondria and a skeletal muscle biopsy was recommended for investigation of a possible respiratory chain deficiency. Unfortunately deterioration in clinical condition, with worsening heart failure, precluded the possibility of an invasive diagnostic procedure. She required increasing inotropic support and developed a profound lactic acidosis eventually succumbing to heart failure at the age of 4 months.

A male cousin of the index case, born to non-consanguineous parents, had an older sibling who died from cardiomyopathy. Postnatal cardiac screening of this infant revealed biventricular HCM and 3 cm hepatomegaly was noted. Tone was generally low and he was considered slightly delayed across all developmental domains assessable at 6 months. Blood lactate was marginally elevated but urine organic and plasma amino acids were reported normal. At the

age of 7 months he developed seizures and antiepileptic medication was commenced with good effect. Cardiomyopathy progressed and he developed complete heart failure that was not amenable to treatment. In view of likely neurological involvement, he was not considered for cardiac transplant.

Patient 6
c.798-1G>T; c.1690C>A consensus splice mutation; p.Arg564Ser

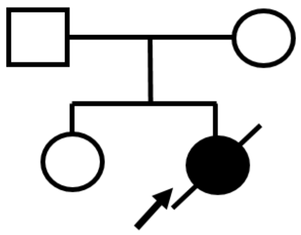

Patient 6 was the daughter born to non-consanguineous Italian parents; an older healthy sister is now 4 years old. She was born at term at 36 weeks of gestation with a normal weight for gestational age (2.8 kg). She was admitted to the hospital at 3 months of age for developmental arrest and feeding problems with recurrent vomiting. Arterial blood gas revealed severe metabolic acidosis (lactate 15 mmol/L, NR <2 mmol/L). ECG and echocardiography showed ventricular dysfunction on the left side (ejection fraction <30%); serum analyses for myocarditis were negative. Electromyography was normal, as well as liver function tests. Tracheal intubation and mechanical ventilation were required. After a worsening of her cardiac conditions, a LV assist device (LVAD) was implanted. A diagnostic muscle biopsy revealed evidence of an isolated mitochondrial complex I deficiency, although studies in cultured skin fibroblasts appeared normal. She died at age 5 months from respiratory failure.

A mitochondrial disease gene panel revealed a paternally-inherited c.1690C>A, p.Arg564Ser variant and a maternally-inherited c.798-1G>T splicing mutation, shown by cDNA analysis to cause nonsense-mediated decay. The unaffected sister is a heterozygous carrier of the c.798-1G>T variant.

Patient 7
c.1979A>T; c.2039C>T (p.Lys660Ile; p.Ala680Val)

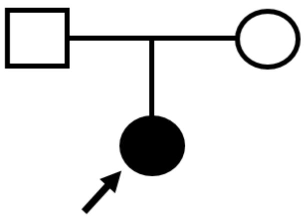

The extensive clinical details of Patient 7 have previously been published by Santorelli and colleagues [2]. Following heart transplantation at age 3.8 years, this female patient is now in adulthood, showing good heart compensation and is on chronic cyclosporine treatment. She complains of mild muscle fatigability, and a stress test has confirmed a moderate exercise intolerance. Blood lactate levels were elevated (4.5 mmol/L; NR 0.7-2.1 mmol/L). Liver and renal function tests are normal, and serial examinations have not revealed an organic aciduria. She has a slight intellectual disability but is independent in her daily life, living at home with her parents. Whole exome sequencing identified segregating *ELAC2* variants; a maternally-inherited c.1979A>T, p.Lys660Ile variant and a paternally-inherited c.2039C>T, p.Ala680Val variant.

Patient 8
c.245+2T>A; c.1264C>G (consensus splice mutation; p.Leu422Val)

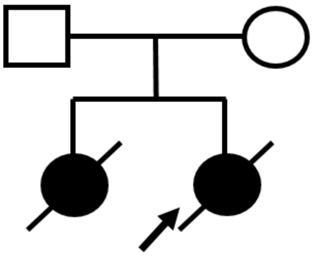

Patient 8 was the second daughter born to healthy, unrelated Italian parents. She was born at term following an uneventful pregnancy that included repeated fetal echocardiography, which was always revealed within normal heart parameters. She was admitted to the hospital at 2 months of age for acute decompensated heart failure, with marked hypokinesis of the left ventricle (EF 30%). She died at 3 months of age; muscle samples taken at autopsy revealed an isolated defect of mitochondrial complex I activity, although this was normal in cultured skin fibroblasts. An older sister had died at 10 months of age with a dilated cardiomyopathy; she was born at term, presenting at 4 months with feeding difficulties, repeated vomiting and diarrhoea. Echocardiography revealed cardiomegaly and concentric LV wall thickening with decrease ejection fraction (EF 25%). Absence seizures were witnessed during neurological examination and optic nerve atrophy was also noted. A cardiac biopsy showed an isolated mitochondrial complex I deficiency but a limited genetic screen for pathogenic variants in mitochondrial DNA, *ACAD9* and *TMEM70* failed to detect the underlying molecular abnormality. Subsequently WES in Patient 8 identified segregating *ELAC2* variants; a maternally-inherited c.1264C>G, p.Leu422Val variant and a paternally-inherited c.245+2T>A splice site mutation, leading to (experimentally-confirmed) exon skipping.

Patient 9
c.1163A>G; c.1163A>G (p.Gln388Arg; p.Gln388Arg)

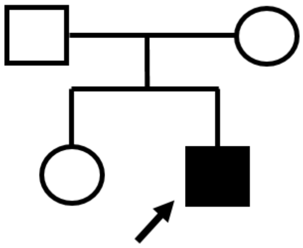

Patient 9 is the second child, now aged 19 years, born to healthy, unrelated Italian parents. He has a healthy older sister. At six months of age, he presented with vomiting, poor feeding, failure to thrive and psychomotor retardation. He walked alone at 2 years and showed delayed speech, muscle weakness and exercise intolerance. His growth was impaired (weight and height <3rd centiles). At 9 years, endocrinological investigations were normal but a cardiac evaluation by echocardiography showed a LVH with normal function. Metabolic screening showed normal lactate, acylcarnitines and organic acids but plasma alanine was increased. Brain MRI demonstrated only non-specific alteration of the periventricular white matter: H-MR spectroscopy was normal. EMG/ENG showed axonal neuropathy. A diagnostic skeletal muscle biopsy revealed a severe and isolated defect of mitochondrial complex I activity. He was treated with riboflavin (50 mg x3/day), L-carnitine (500 mg/day), thiamine (150 x2/day). He showed progressive cardiac impairment, with LV dilatation and hypokinesia (EF 15-20%) at last evaluation (18 years of age). He is currently awaiting cardiac transplantation.

Genetic investigations excluded pathogenic variants in mitochondrial DNA, *ACAD9*, *MTO1* and *TMEM70*. A mitochondrial disease gene panel identified a homozygous c.1163A>G, p.Gln388Arg *ELAC2* variant; both parents were confirmed to be heterozygous carriers.

Patient 10
c.457delA; c.2342G>A (p.Ile153Tyrfs*6; p.Arg781His)

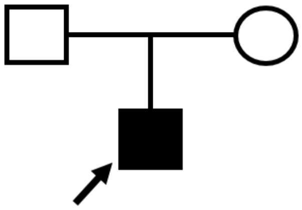

Patient 10 is the first son of healthy, unrelated Caucasian parents of Eastern European descent (Polish). He presented at 8 months of age with severe hypertrophic and dilated cardiomyopathy, initially managed with Berlin heart (ventricular assist device) and extra-corporeal membrane oxygenation (ECMO) prior to cardiac transplant at 10 months of age [3]. He has subsequently developed gastro-intestinal dysmotility and bone marrow failure, which was initially consistent with pure red cell aplasia due to parvovirus infection, but evolved to affect all cell lines, suggesting ineffective hematopoiesis secondary to a mitochondrial defect. He has hypotonia and global developmental delay. He is currently 6 years old, attends mainstream school, sits independently and is walking with assistance. Family history was unremarkable with the exception of prostate cancer in several paternal relatives. Electron microscopy of a cardiac biopsy revealed an increased number of abnormal swollen mitochondria with bizarre morphology including vacuoles, abnormal cristae and rod-like profiles. Assessment of mitochondrial respiratory chain activities in cultured skin fibroblasts showed a defect in the rotenone-sensitive, NADH:cytochrome *c* reductase (complex I+III) assay (<40% of control). WES identified segregating *ELAC2* variants; a paternally-inherited c.457delA, p.Ile153Tyrfs*6 variant and a maternally-inherited c.2342G>A, p.Arg781His variant.

Patient 11
c.460T>C; c. 460T>C (p.Phe154Leu; p.Phe154Leu)

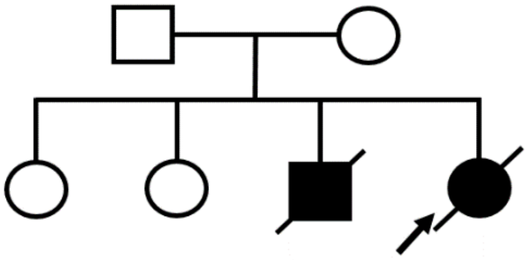

Patient 11 was the fourth child of healthy unrelated Kuwaiti-Arab parents. She was born by elective caesarean section at 37 weeks gestation following an uneventful pregnancy, with a birth weight of 2.5 kg. Two older sisters are well but an older brother had died at the age of 2.5 months of cardiomyopathy associated with lactic acidosis. In view of the family history, she was admitted to the special care baby unit at birth and shown to have an elevated blood lactate at 4.5 mmol/L (NR 0.7-2.1 mmol/L). Blood lactate levels continued to fluctuate between 1.2 and 3.8 mmol/L. An initial echocardiogram revealed possible mild pulmonary stenosis but no evidence of cardiomyopathy. Physical examination revealed an alert, active baby with no evidence of cardiac compromise. Serial echocardiography demonstrated progressively worsening LVH. At 7 weeks she had concentric LVH with ejection fraction of 32%, interventricular septal thickness of 9mm (normal 3.5-4.8mm) and posterior wall thickness of 6mm (normal 3.5-5.0mm). Electrocardiograms showed progressively worsening LVH. CSF lactate was normal at 1.3 mmol/L with an elevated CSF protein of 0.95 g/L. Skeletal muscle biopsy revealed lipid storage myopathy but no other mitochondrial histopathological changes; assessment of mitochondrial respiratory chain enzyme activities showed an isolated complex I deficiency. She became more unwell with symptoms of heart failure and worsening lactic acidosis (maximum blood lactate 15.0 mmol/L) and died of cardiac failure at 2.5 months.

A previously-reported, homozygous p.Phe154Leu *ELAC2* variant was identified following WES; parents were confirmed to be heterozygous carriers.

Patient 12
c.2245C>T; c. 297-2_297-1delinsT (p.His749Tyr; consensus splice mutation)

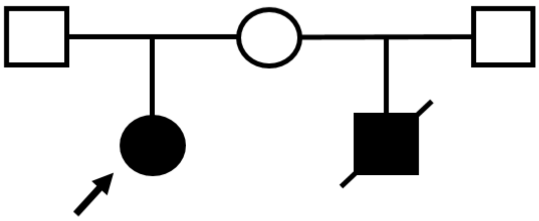

Patient 12 is a daughter born to healthy, unrelated parents of African-American ethnicity following an uneventful pregnancy. A half-brother died in infancy awaiting cardiac transplant. She presented at 1 month of age with a strikingly hypertrophic, biventricular cardiomyopathy and severe lactic acidosis. At 4 months of age, she developed respiratory failure and pericardial effusion, first managed with ECMO then heart transplant at the age of 6 months. She developed acute graft failure, received a second heart transplant at 10 months old, but continued to have respiratory failure later developing renal failure. Brain imaging revealed diffuse volume loss. Following two cardiac transplants complicated by graft failure, she died at 13 months old from enterococcus sepsis and CMV viremia with colitis. Autopsy revealed involvement of liver, with bile ductular proliferation, early portal, pericentral and marked sinusoidal fibrosis and centrilobular congestion; hepatocytes appeared to show mitochondrial proliferation. In addition, there was early, acute tubular necrosis in kidneys and mild thickening of the bowel wall with prominent myenteric plexus.

A diagnostic muscle biopsy performed at 4 months of age showed myofibre atrophy, multiple mitochondrial respiratory chain deficiencies involving complexes I, III and IV. Electron microscopy revealed mild alteration of mitochondrial cristae; in contrast, explanted cardiac tissue showed a vast excess of mitochondria displaying abnormal morphology.

WES identified segregating *ELAC2* variants; a paternally-inherited c.297-2_297-1delinsT mutation predicted to disrupt the splice acceptor in intron 3 and a maternally-inherited c.2245C>T, p.His749Tyr variant.

Patient 13
c.460T>C; c. 460T>C (p.Phe154Leu; p.Phe154Leu)

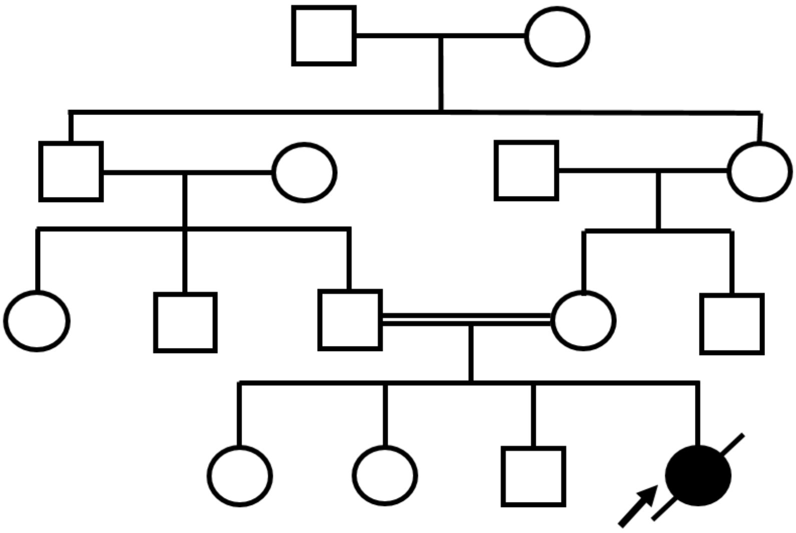

Patient 13 was the fourth female child of healthy, consanguineous, first cousin Saudi Arabian parents. She was born at term with a birth weight of 2.1 kg (<5th centile), birth length of 46 cm (<5th centile) and head circumference of 31.5 cm (<5th centile). Failure to thrive was diagnosed at the age of 5 months with weight, length and head circumference all remaining below the 5th centile. She was also noted to have a severe HCM with elevated levels of serum lactate recorded (3 mmol/L, NR < 2.2 mmol/L). Neurological examination showed poor head control. Plasma amino acids showed elevated alanine, whilst urinary organic acids, transferrin iso-electrofocussing, very long chain fatty acids, chromosomal analysis, eye examination and hearing test were all normal. Echocardiogram revealed a severe, concentric LVH. She was admitted to hospital but died two days later despite full support.

Targeted genetic studies excluded pathogenic variants in the *GAA* and *ACADVL* genes. A previously-reported, homozygous p.Phe154Leu *ELAC2* variant was identified following WES; parents were confirmed to be heterozygous carriers.

Figure S1 | Protein expression and pre-tRNA processing kinetics with mitochondrial tRNA$^{Leu(UUR)}$ substrate

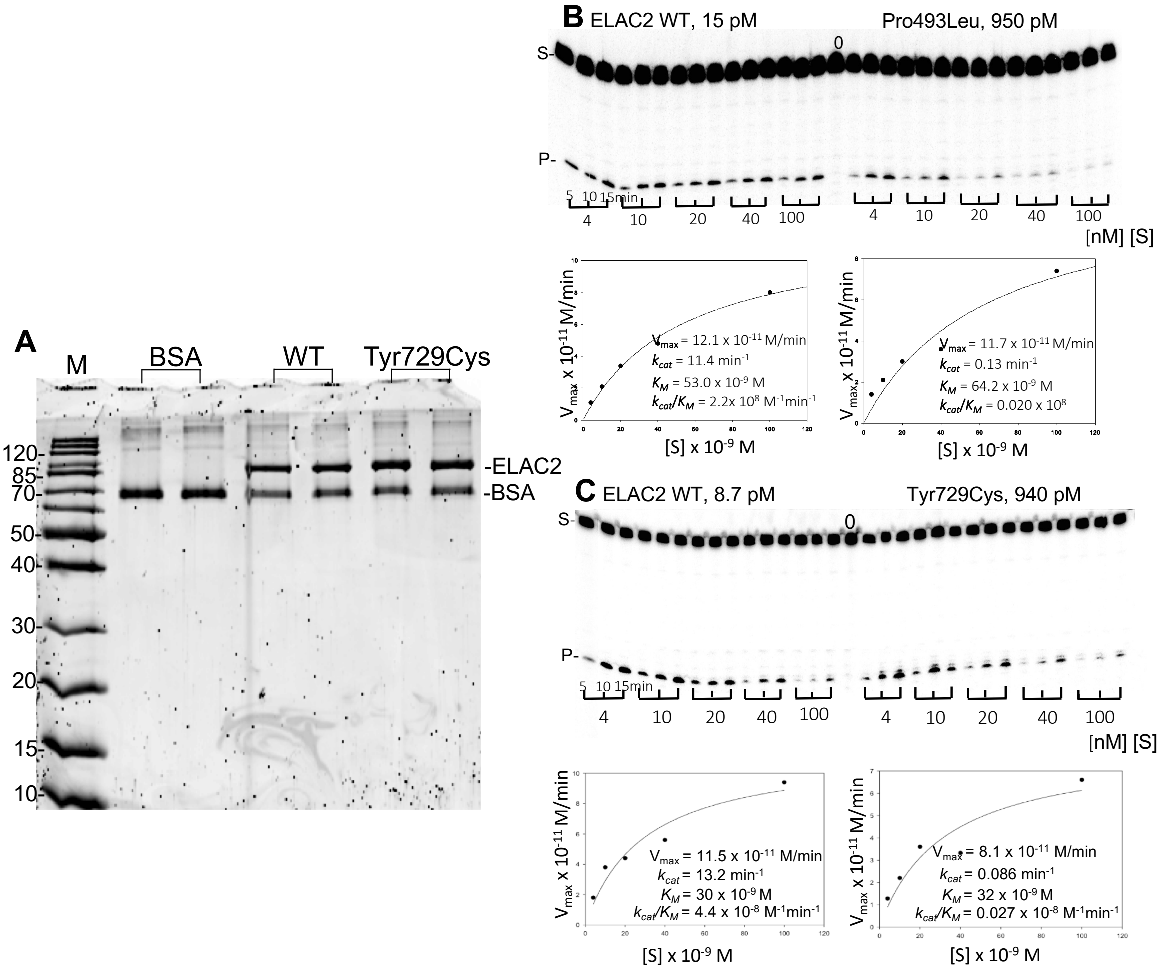

(A) Wild type (WT) and mutant proteins were electrophoresed on SDS polyacrylamide gels to assess purity and mass load. BSA was used as a mass standard. The protein standard (M) is the unstained recombinant protein ladder. Experimental lanes were loaded in duplicate. Protein concentrations and sample volumes were adjusted to an intended load of 500 ng. BSA in tRNase Z lanes was used as a carrier in the processing reaction buffer to help preserve enzyme activity in the dilution series (to a final enzyme concentration of ~10 pM for wild type reactions). Protein concentrations in processing reactions (see B and C) were determined from corrected values obtained by comparison with band intensities in the BSA lanes.

(B-C) Mutations in ELAC2 analysed in this work were expressed using baculovirus, affinity purified and used in processing kinetic experiments. In this example, kinetic experiments were performed using WT and Pro493Leu **(B)** or WT and Tyr729Cys (**C**) enzymes at the indicated concentrations with a constant concentration of 5' end-labelled pre-tRNA$^{Leu(UUR)}$, varying the concentration of unlabelled substrate as indicated below the panels, covering a range from 4 – 100 nM [S]. Reactions were sampled after 5, 10 and 15 min. Analysis was performed by electrophoresis on 6% denaturing polyacrylamide gels which were dried, exposed using imaging screens, and scanned with a Typhoon 9410 imager. Lanes from the gel were analyzed using Imagequant and interpreted using Sigmaplot. Intended [E] and [S] in the experiments were adjusted using analytical protein gel lanes and analytical RNA gel lanes (not shown). % product/min reaction, which clearly decreases with increasing [S], is V/[S]. This value converts to V when multiplied by unlabelled [S], producing the asymptotic plots. Units for *kcat*, K_M, k_{cat}/K_M in the mutant experiment are the same as for WT.

Figure S2 | Analysis of unprocessed mitochondrial tRNA-mRNA intermediates

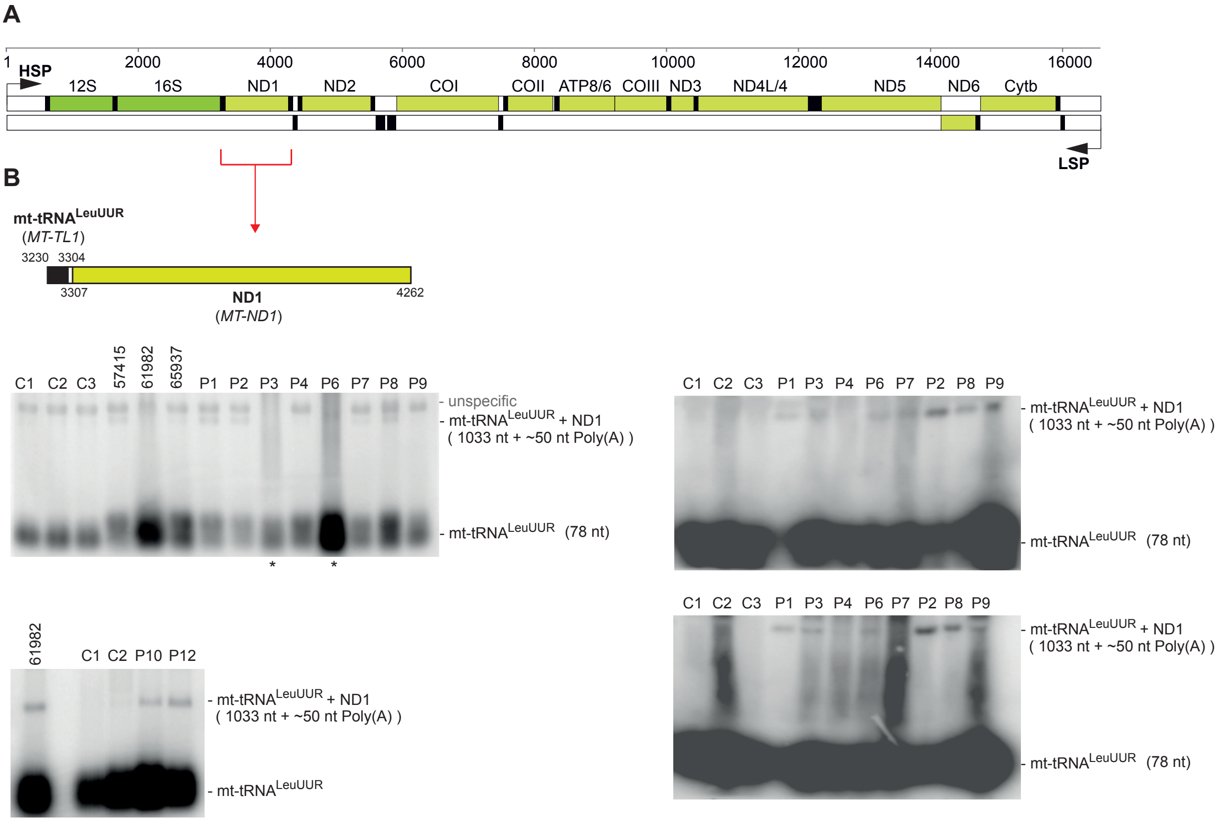

(A) Linear genetic map of mtDNA (numbering according to RefSeq accession number J01415) indicating mt-rRNA (green), mt-mRNA (olive) and mt-tRNA (black). Non-coding sequences in white. LSP – Light strand promoter. HSP – Heavy strand promoter. The unprocessed mt-tRNA$^{Leu(UUR)}$-ND1 mRNA unit is indicated by red brackets.

(B) Northern blot processing analysis of the mt-tRNA$^{Leu(UUR)}$-ND1 mRNA junction in total RNA samples of control fibroblasts (C1-C3), fibroblasts from the previously published cases (57415, 61982, 65937)[4] and fibroblasts from the patients harbouring novel ELAC2 mutations (P1-4, P6-9 and P12). Asterisks indicate partially degraded RNA samples that were reanalysed in a different blot and presented in the same panel.

Figure S3 | Multiple sequence alignment of eukaryotic ELAC2 proteins

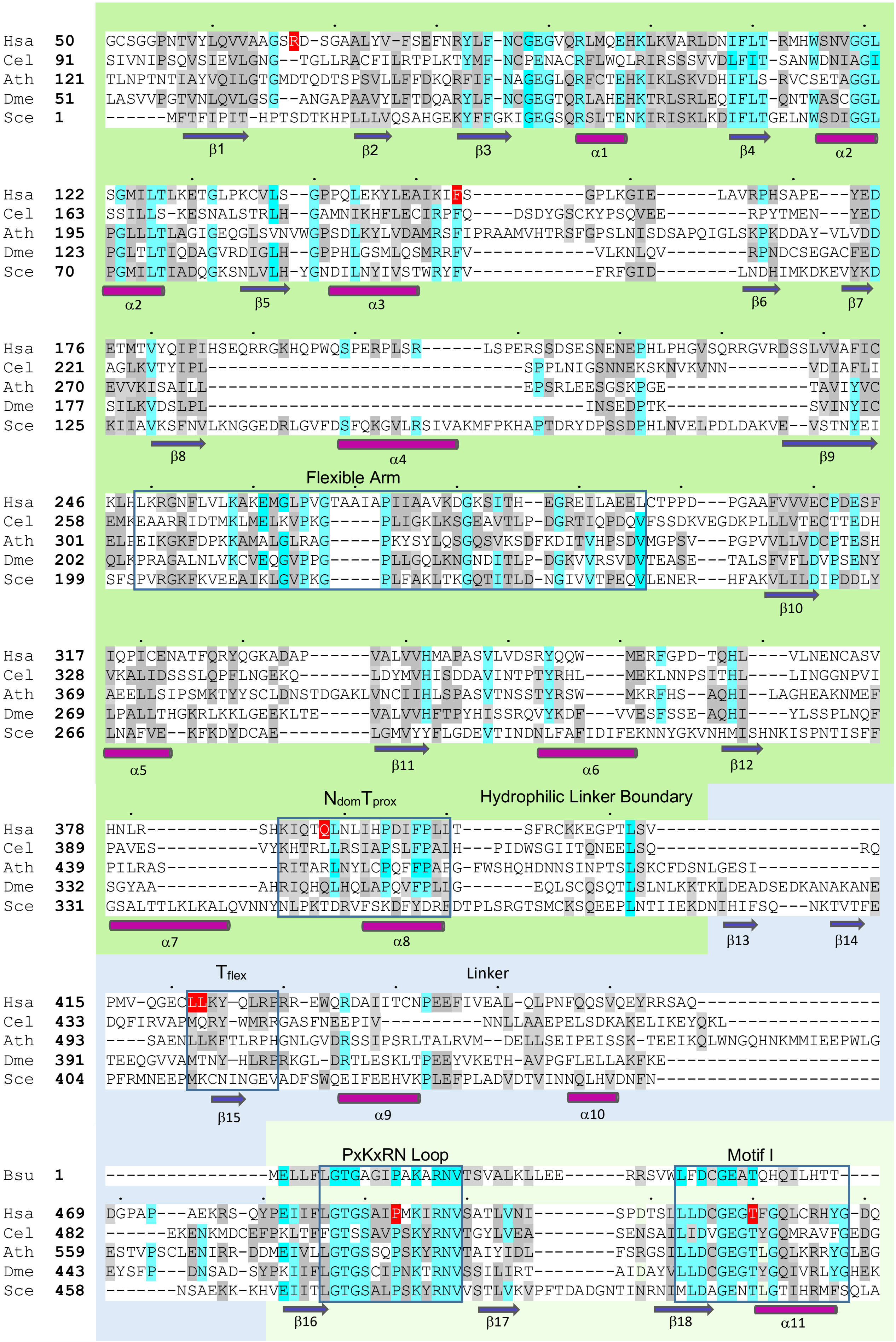

Figure S3 | Multiple sequence alignment of eukaryotic ELAC2 proteins - continued

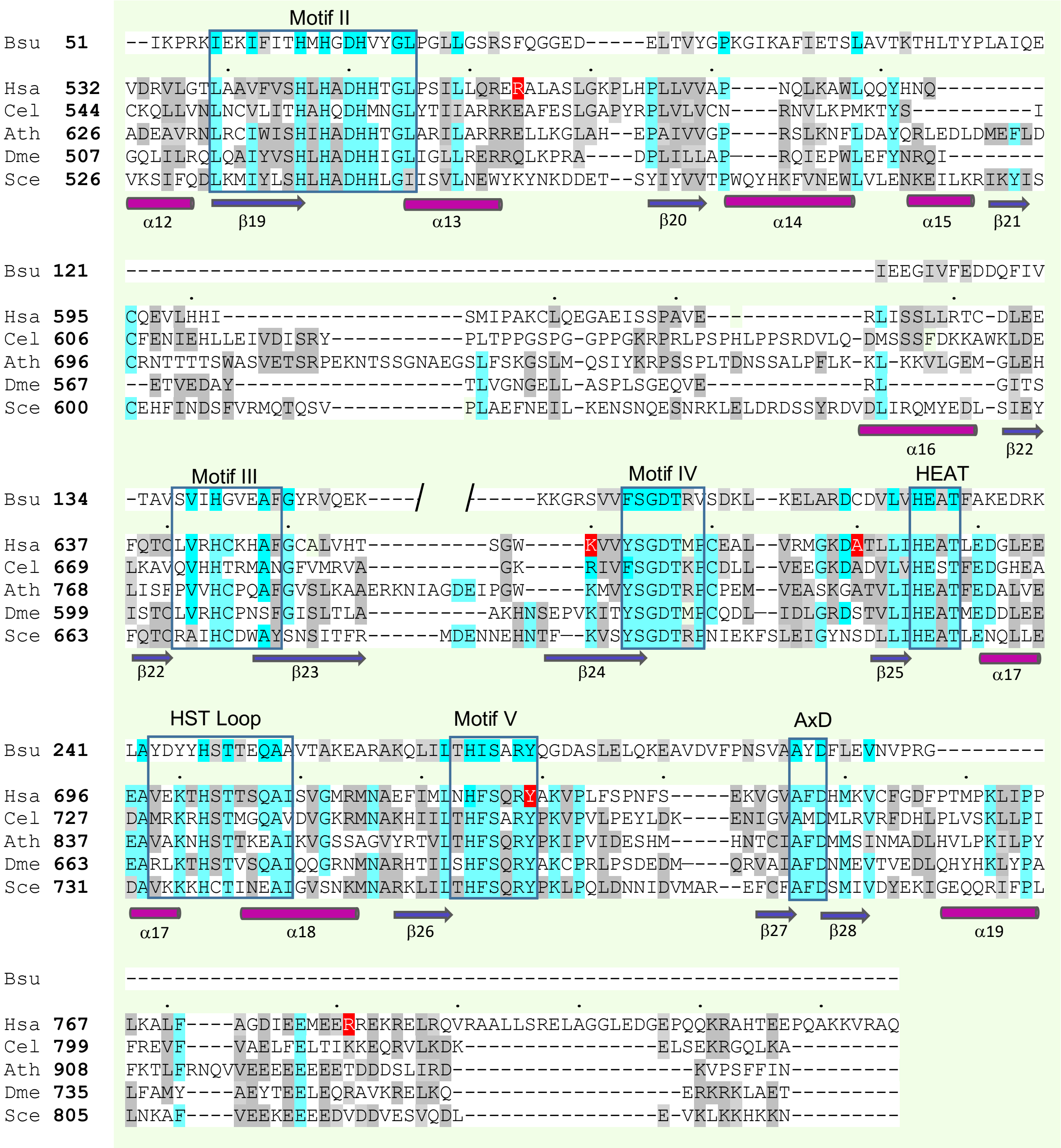

Multiple sequence alignment of five eukaryotic ELAC2 orthologues: *H.sapiens* (Hse, NP_060597.5), *C.elegans* (Cel, NP_001023109.1), *A.thalania* (Ath, NP_188247.2), *D.melanogaster* (Dme, NP_724916.1), and *S.cerevisiae* (Sce, NP_013005.1) was performed using the EMBL-EBI alignment tool MUSCLE with manual adjustments. The sequence of amino domain, inter-domain linker and carboxy domain are shown on green, blue and pale green background, respectively. Additionally, *B.subtilis* tRNase Z (Bsu) is shown aligned with the carboxy domains of the eukaryotic enzymes with which it shares the greatest homology. The flexible arm of *B.subtilis* tRNase Z corresponding to residues between Motifs III and IV is deleted from this alignment (/K_{152} - K_{200} /). The residues in *H. sapiens* ELAC2 found to be associated with cardiac hypertrophy are highlighted in red. Conserved residues are highlighted in teal and chemically related residues are highlighted in grey. The β-strands and α-helices in *S. cerevisiae* Trz1[5] are shown with ( ) and ( ), respectively.

Figure S4 | Examining mitochondrial disease-related Arg68 and Gln388 residues in human ELAC2 using *S. cerevisiae* Trz1

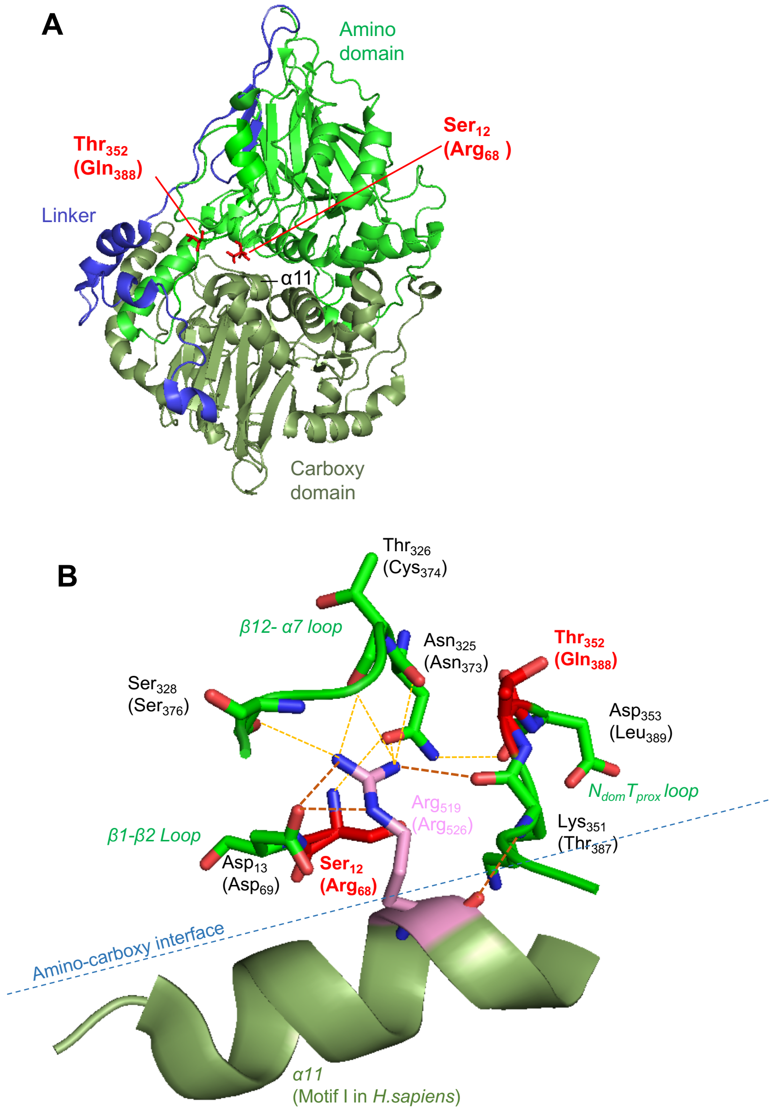

(A) Yeast Trz1 Ser12 and Thr352, corresponding to human Arg68 and Gln388, respectively, are amino domain residues located near the interface between the amino and carboxy domains. Although neither of these residues are conserved in *S. cerevisiae*, there is a high degree of regional conservation. Ser12 occurs in a short loop between highly conserved β1-β2. Thr352 is located in a loop of the conserved region $N_{dom}T_{prox}$ directly preceding linker.

(B) *S. cerevisiae* residues of interest are shown in stick form and labeled, with corresponding *H. sapiens* residues in parentheses. Ser12 and Thr352 (both shown in red) are in the vicinity of a complex network of polar contacts between residues in the amino domain and the highly conserved residue Arg519 (mauve, Arg526 in *H. sapiens*) in α11 of the carboxy domain. Residues adjacent to Ser12 and Thr352 participate directly in this inter-domain web of polar contacts. Further multiple possible polar contacts between the side chains of Arg519 and Asp13 (both conserved), which is next to Ser12 are indicated. Additionally, both the side chain and backbone of Arg519 contact the backbone of Lys351, neighboring Thr352. The model suggests the *H. sapiens* substitutions at Arg68 and Gln388 could disrupt the structure and folding of ELAC2 through their indirect effects on this complex of polar contacts across the domain interface.

Figure S5 | Examining mitochondrial disease-related Phe154 in human ELAC2 using *S. cerevisiae* Trz1

A

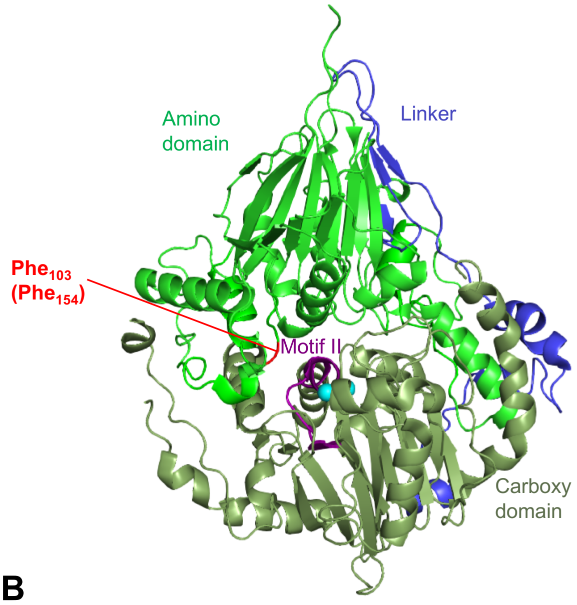

B

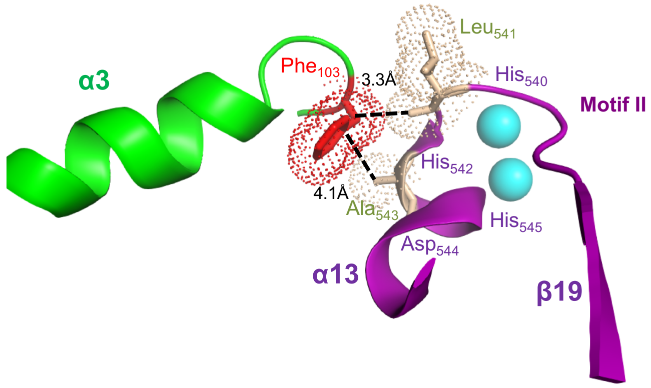

(A) *S. cerevisiae* Trz1 Phe103, corresponding to human Phe154, is located in the α3-β6 loop (mauve). This amino domain residue at the domain interface faces conserved Motif II (HxHxDH) in the β19-α13 loop (magenta) which is directly involved in metal ion binding and catalysis.

(B) Expanded view illustrating a possibility of *S. cerevisiae* Phe103 making hydrophobic contact with the conserved Leu542 in Motif II, and also approaches Ala543, the other conserved hydrophobic residue in this otherwise very hydrophilic region of Motif II. The HxHxDH residues characteristic of all tRNase Zs, are labeled. Divalent metal ions are indicated with blue spheres.

Figure S6 | Examining mitochondrial disease-related Leu422 and Leu423 residues in human ELAC2 using *S. cerevisiae* Trz1

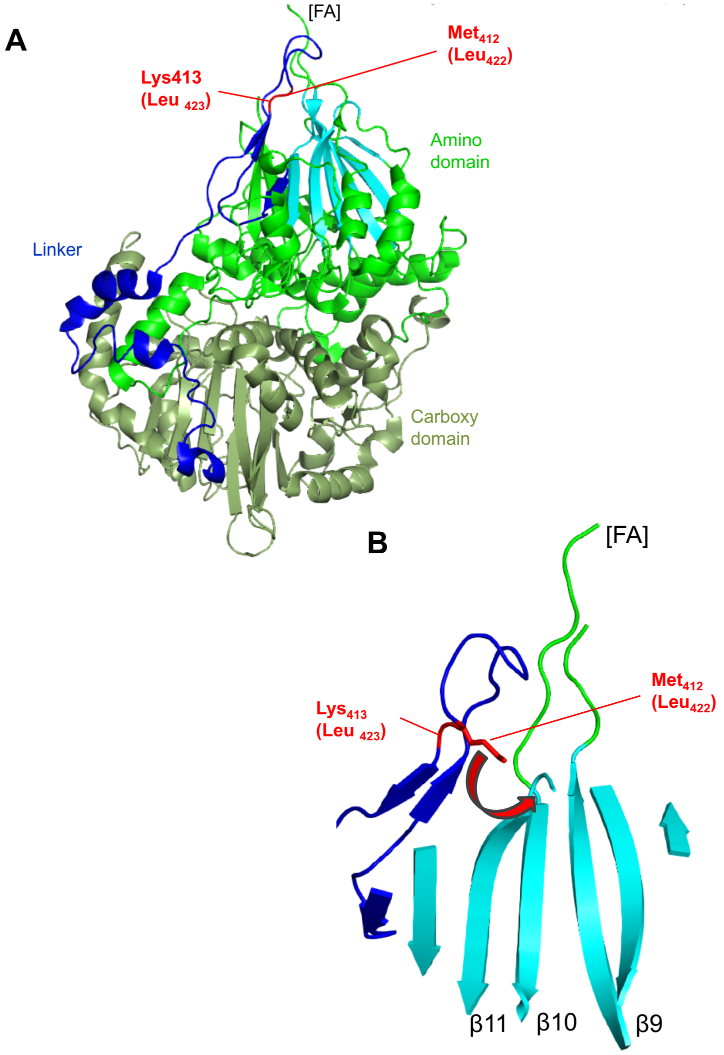

(A) Two of the human mitochondrial disease-associated mutations occur in residues found in the linker between the amino and carboxy domains of eukaryotic ELAC2. Using the crystal structure of *S.cerevisiae* Trz1 (5MTZ), Met412 (*H. sapiens* Leu422) and Lys423 (*H. sapiens* Leu423) are indicated in red. Two twisted β-sheets provide the framework for the amino domain. One of these residues (Met412) is near the second twisted β-sheet (cyan) which includes the β-strands that lead to and from the flexible arm (FA).

(B) Residues corresponding to Leu422 and Leu423 in *H. sapiens* ELAC2 are not conserved in *S. cerevisiae* tRNase Z. Nonetheless, *S. cerevisiae* Met412 (equivalent in position and similar in hydrophobicity to *H. sapiens* Leu422) can be used to model the effects of hydrophobicity changes at these positions. Met412 is at the apex of β10-11, two parallel β-strands in the second twisted β-sheet in the amino domain of ELAC2. The flexible arm that binds the pre-tRNA substrate emerges from the same β-sheet between β9-10. The subtle local hydrophobicity changes in *H. sapiens* missense substitutions Leu422Val and Leu423Phe could be regionally propagated to influence structure of this β-twisted sheet and possibly the flexible arm. Met412 in *S. cerevisiae* tRNase Z (5MTZ, shown) appears to make the most direct hydrophobic contact with the amino end of β10 in the second twisted β sheet in the amino domain, which is also on the descending side of the flexible arm (FA).

Figure S7 | *H. sapiens* ELAC2 mitochondrial disease-related missense substitution Thr520Ile modeled using *S. cerevisiae* Trz1 (5MTZ)

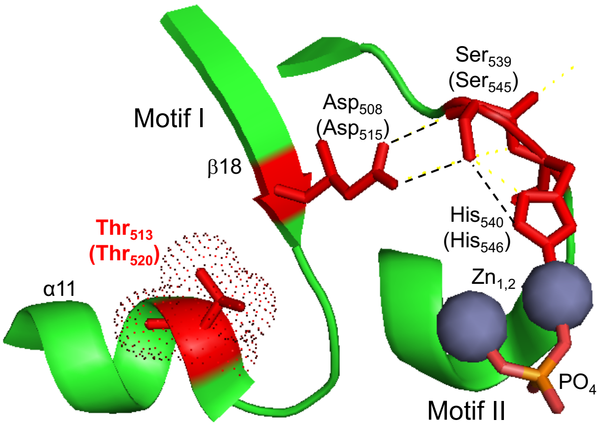

S. cerevisiae Trz1 (5MTZ) model of the Motif I – Motif II region includes Thr513 (Thr520 in *H. sapiens,* at which the *H. sapiens* pathogenesis-related substitution Thr520Ile was observed). The β18-α11 (Motif I) loop includes Asp508 (Asp515 in *H. sapiens*) and Thr513 (Thr520 in *H. sapiens*). Asp508, the Motif I aspartate, initiates a charge relay (dashed lines indicate polar contacts) through Ser539 (Ser545) to His540 (His546). $Zn_{1,2}$ are metal ions required for catalysis and PO_4 is a phosphate that mimics the scissile bond. Dots shown surrounding Thr513 emphasize hydrophobicity, which increases with the *H. sapiens* pathogenesis-related substitution Thr520Ile.

Figure S8 | *H. sapiens* ELAC2 mitochondrial disease-related missense substitution at Lys660, Ala680, and His749 modeled using *S. cerevisiae* Trz1 (5MTZ)

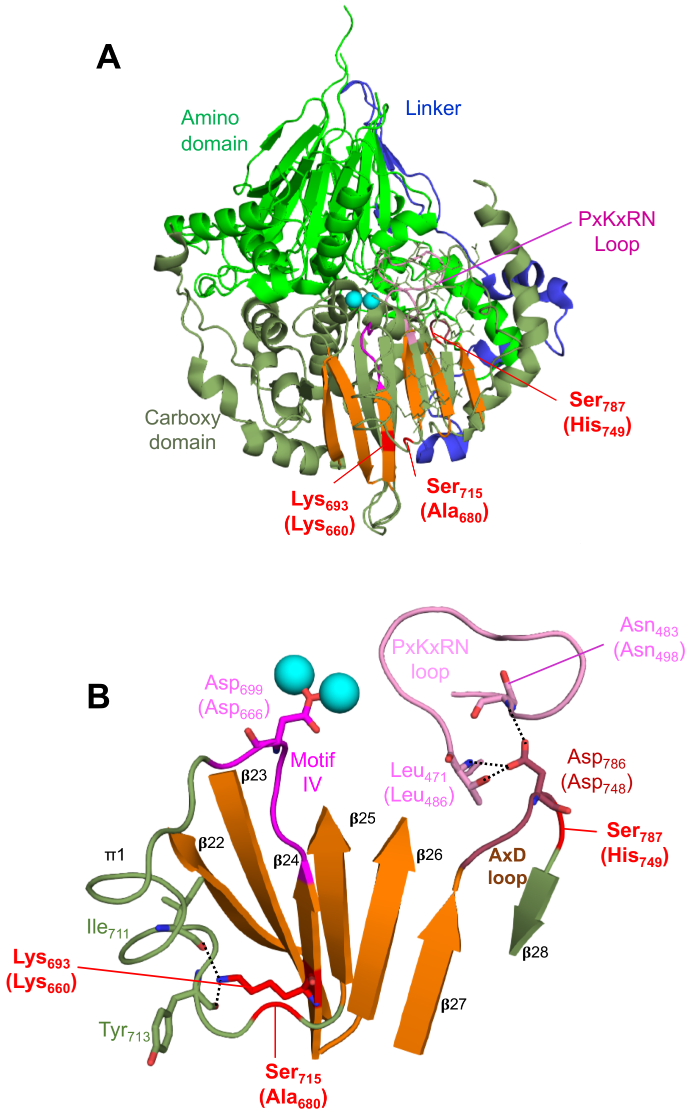

(A) The *S. cerevisiae* Trz1 structure 5MTZ with color coding as in previous figures. Additionally, a **β** twisted sheet in the carboxy domain (**β**22 - **β**27) is indicated in orange, the Motif IV loop is in violet, the region following the Motif IV loop is in green, the AxD loop is shown in brown and the PxKxRN loop is inpink. Lys693 (Lys660), Ser715 (Ala680) and Ser787 (His749) are labelled in red (residues listed for *S. cerevisiae* Trz1 and in brackets for *H. sapiens* ELAC2.

(B) An expanded view emphasizing the relationship between Lys693 (Lys660) and Ser715 (Ala680) and Motif IV and between Ser787 (His749) and the AxD loop. Selected residues shown as sticks and polar contacts are illustrated with bold dashed lines.

Figure S9 | *H. sapiens* ELAC2 mitochondrial disease-related missense substitution Arg781His modeled using S. cerevisiae Trz1 (5MTZ)

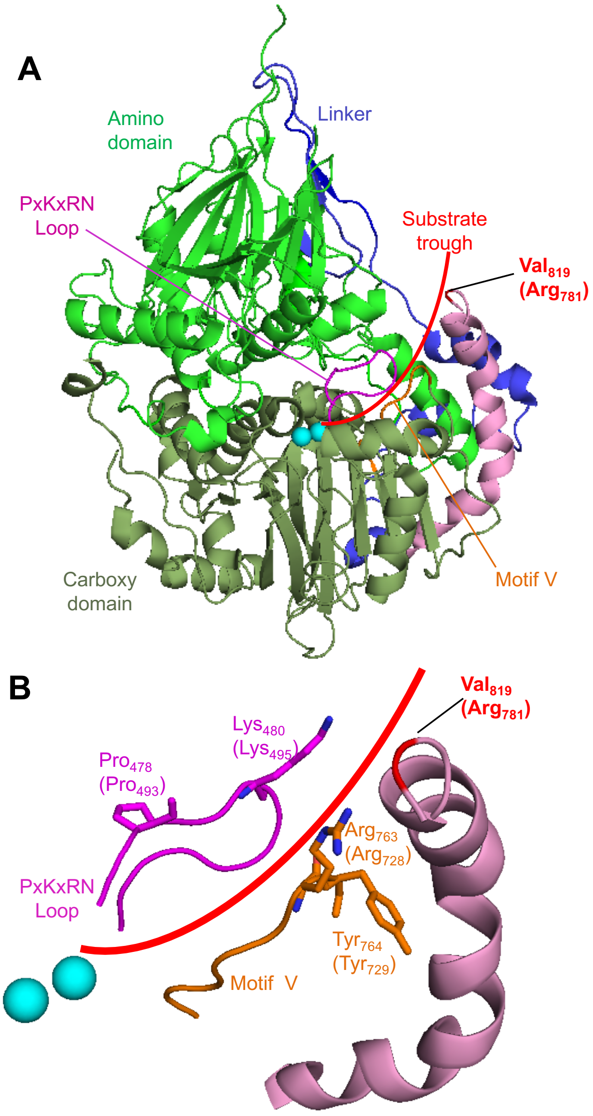

(A) Structure of *S. cerevisiae* Trz1. Amino and carboxy domains and linker are colored as in previous figures. Eukaryotic ELAC2 enzymes have a long C-terminal alpha helix (pink, on right) which is absent from tRNase ZSs (ELAC1) (see alignments). *H. sapiens* Arg781 is equivalent in position to the second to last structured residue in *S. cerevisiae* tRNase Z. Red arc indicates the presumed substrate path (effectively modeled in **Figure 5** using the *B. subtilis* enzyme-substrate co-crystal structure). Metal ions which mark the active site are shown as blue spheres. PxKxRN and Motif V loops are shown in magenta and orange, respectively.

(B) Detailed view of the *S. cerevisiae* tRNase Z long α-helix (pink) approaching the predicted location where substrate acceptor stem is clamped by polar contacts between Lys in the PxKxRN loop (Lys495 in *H. sapiens*, Lys480 in *S. cerevisiae*, Lys15 in *B. subtilis*) and nt +1-2 of the pre-tRNA substrate and between Arg in the Motif V loop (Arg728 in *H. sapiens*, Arg763 in *S. cerevisiae*, Arg274 in *B. subtilis*) and nt 71-72-73 of the substrate (**Figure 5**). Metal ions of the active site are shown as blue spheres. PxKxRN and Motif V loops are shown in magenta and orange, respectively. Red arc indicates the presumed substrate path.

SUPPLEMENTARY REFERENCES

1. Taylor RW, Pyle A, Griffin H, Blakely EL, Duff J, He L, et al. Use of whole-exome sequencing to determine the genetic basis of multiple mitochondrial respiratory chain complex deficiencies. JAMA. 2014;312(1):68-77. doi: 10.1001/jama.2014.7184. PubMed PMID: 25058219.
2. Santorelli FM, Gagliardi MG, Dionisi-Vici C, Parisi F, Tessa A, Carrozzo R, et al. Hypertrophic cardiomyopathy and mtDNA depletion. Successful treatment with heart transplantation. Neuromuscul Disord. 2002;12(1):56-9. PubMed PMID: 11731286.
3. Parikh S, Karaa A, Goldstein A, Ng YS, Gorman G, Feigenbaum A, et al. Solid organ transplantation in primary mitochondrial disease: Proceed with caution. Mol Genet Metab. 2016;118(3):178-84. doi: 10.1016/j.ymgme.2016.04.009. PubMed PMID: 27312126.
4. Haack TB, Kopajtich R, Freisinger P, Wieland T, Rorbach J, Nicholls TJ, et al. ELAC2 mutations cause a mitochondrial RNA processing defect associated with hypertrophic cardiomyopathy. Am J Hum Genet. 2013;93(2):211-23. doi: 10.1016/j.ajhg.2013.06.006. PubMed PMID: 23849775; PubMed Central PMCID: PMC3738821.
5. Ma M, Li de la Sierra-Gallay I, Lazar N, Pellegrini O, Durand D, Marchfelder A, et al. The crystal structure of Trz1, the long form RNase Z from yeast. Nucleic Acids Res. 2017;45(10):6209-16. doi: 10.1093/nar/gkx216. PubMed PMID: 28379452; PubMed Central PMCID: PMCPMC5449637.
